# Extracellular Matrix- and Integrin Adhesion Complexes-Related Genes in the Prognosis of Prostate Cancer Patients’ Progression-Free Survival

**DOI:** 10.3390/biomedicines11072006

**Published:** 2023-07-15

**Authors:** Ivana Samaržija, Paško Konjevoda

**Affiliations:** Laboratory for Epigenomics, Division of Molecular Medicine, Ruđer Bošković Institute, 10000 Zagreb, Croatia; pasko.konjevoda@irb.hr

**Keywords:** prostate cancer, extracellular matrix, integrin adhesion complex, cancer prognosis, progression-free survival, recursive partitioning, Gleason score, FMOD, PTPN2

## Abstract

Prostate cancer is a heterogeneous disease, and one of the main obstacles in its management is the inability to foresee its course. Therefore, novel biomarkers are needed that will guide the treatment options. The extracellular matrix (ECM) is an important part of the tumor microenvironment that largely influences cell behavior. ECM components are ligands for integrin receptors which are involved in every step of tumor progression. An underlying characteristic of integrin activation and ligation is the formation of integrin adhesion complexes (IACs), intracellular structures that carry information conveyed by integrins. By using The Cancer Genome Atlas data, we show that the expression of ECM- and IACs-related genes is changed in prostate cancer. Moreover, machine learning methods revealed that they are a source of biomarkers for progression-free survival of patients that are stratified according to the Gleason score. Namely, low expression of *FMOD* and high expression of *PTPN2* genes are associated with worse survival of patients with a Gleason score lower than 9. The *FMOD* gene encodes protein that may play a role in the assembly of the ECM and the *PTPN2* gene product is a protein tyrosine phosphatase activated by integrins. Our results suggest potential biomarkers of prostate cancer progression.

## 1. Introduction

Prostate cancer is among the most common cancers with regard to incidence and mortality [1,2]. According to the Global Cancer Observatory, in 2020, there were 1,414,259 new prostate cancer cases diagnosed (7.3% of all sites) and 375,304 deaths from this disease (3.8% of all sites) [3]. Surgical intervention (radical prostatectomy) and radiotherapy are the usual treatment options for localized prostate cancer [4,5]. However, the biochemical recurrence, which is defined by a rise in the blood level of prostate-specific antigen (PSA), occurs within 10 years in a fraction of patients treated with radical prostatectomy (20–40% of cases) and radiotherapy (30–50% of cases) [6]. Biochemical recurrence is usually a sign of a progressive disease, which is accompanied by symptoms or evidence of disease progression on imaging [7]. Although the five- and ten-year survival rates in prostate cancer are favorable in comparison to some other more aggressive cancer types, the recurrence of the disease is fatal for a substantial number of patients. The probability to develop prostate cancer highly increases with age, and it is considered that 30–40% of men older than 50 years of age have prostate cancer, but not all cases are clinically significant [8]. In line with these observations, one of the greatest obstacles in prostate cancer treatment is the inability to foresee the course of the disease and to recognize the tumors that will be indolent and require no or minimal intervention and those that are more malignant and will progress fast. Therefore, novel biomarkers of disease progression and therapeutic targets are needed [9].

Solid cancers, such as prostate cancer, are composed of malignant cells, which are surrounded by a tumor microenvironment consisting of the extracellular matrix (ECM) and the host cells, among which are immune cells, fibroblasts, endothelial cells, adipocytes, stellate cells, and other stromal cells [10]. These host cells are often hijacked by tumor cells to perform the roles that suit the tumor cell growth, proliferation, migration, and other malignant behaviors. The important part of the tumor microenvironment and the first frontier of the cell towards its surroundings is its ECM, a three-dimensional network consisting of extracellular macromolecules and minerals. Collagen is the most abundant ECM protein, constituting up to 90% of the ECM and 30% of the total protein in humans [11]. Other common ECM fibrous proteins are fibronectins, laminins, and elastins. Besides the ECM proteins that give a structure to the tissue, ECM is a rich source of bound growth factors, cytokines, and other secreted proteins that add to the bi-directional communication between tumor and host cells within the tumor microenvironment. Although the ECM was at first considered to be only a plain structural scaffold, later it became more and more clear that it plays active, important roles in cell signaling, properties, and morphology [12]. It is considered that the ECM regulates and fine-tunes every cellular process in the body, and during tumorigenesis it influences tumor onset, progression, and metastatic dissemination [13]. Therefore, the ECM is an interesting and growing topic to investigate, also regarding prostate cancer tumorigenesis [14].

The ECM components such as collagens, laminins, fibronectin, and vitronectin are ligands for integrin transmembrane receptors. Integrins are heterodimers formed of α and β subunits. In humans, there are 18 known α and 8 β subunits, which form 24 known heterodimers [15,16]. Integrins are among the main hubs that link the ECM and the cell interior [17,18]. Their dynamic expression on the cell surface [19] conveys the information about the cell surroundings inside the cell. The corresponding integrin adhesion complexes (IACs) that are formed on a cytoplasmic side upon integrin ligation and activation differ in their appearance, size, and dynamics [20,21]. Consequently, IACs also differ in their protein composition and connections to the intracellular components. For examples of this diversity, see [22,23,24,25]. By using a variety of different downstream entities, the communication between the cell exterior and interior acquires many subtle shades that shape the cell’s response to its surroundings.

In this article, we analyze the prostate cancer ECM gene expression (matrisome) and its changes in comparison to healthy prostate tissue. We extended our analysis on ECM’s integrin receptors and their adhesion complexes (adhesome) to credit the central roles that these proteins play in ECM–cell communication. By analyzing The Cancer Genome Atlas (TCGA) gene expression data, we found evidence that indicates high perturbations in matrisome and adhesome composition in prostate cancer, which we linked to the clinical information. In our previous article [26], we have shown that the Gleason score is the most informative prognostic variable in the analysis of progression-free survival (PFS) in the prostate cancer dataset. However, in this publication, we refined this result by adding the ECM and IAC genes’ expression variables to the analysis. Our results suggest that, among the IACs-related genes, the expression of *PTPN2* further defines the survival tree for patients with a Gleason score lower than 9. The higher expression of the *PTPN2* gene was associated with worse progression-free survival. Additionally, among the ECM-related genes, the expression of the *FMOD* gene further advanced the definition of progression-free survival risk subgroups of patients with a Gleason score lower than 9. The lower expression of the *FMOD* gene was associated with worse survival for those patients.

With this article, we aimed to achieve several goals: (a) to widen the knowledge on potential changes of ECM- and IACs-related genes in prostate cancer, and (b) to propose potential biomarkers for the prognosis of progression-free survival in prostate cancer. These results would hold potential to guide the treatment options for prostate cancer patients. Another value of this paper is methodological since: (c) we used recursive partitioning and survival trees for the establishment of prognostic subgroups. Considering the prostate cancer heterogeneity, we trust that our approach better-describes its characteristics. Additionally, survival trees are easier to interpret and present by clinicians than the Cox regression results.

## 2. Materials and Methods

**Figure 1:**
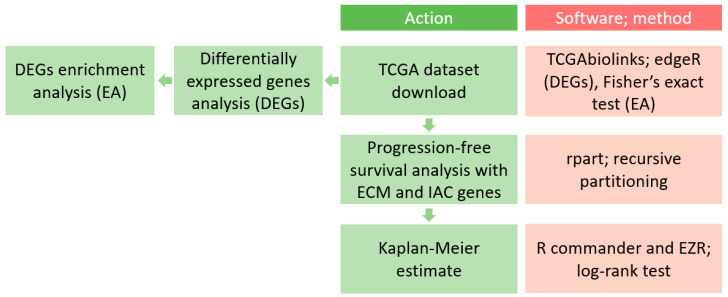
The workflow of this study. The conducted steps are shown in green rectangles. The software used, and the method that each performs, is shown in red rectangles. ECM, extracellular matrix; IAC, integrin adhesion complex; EZR, Easy R.

The main methodological workflow of this article is presented in Figure 1 and described in the following sections. Briefly, after the TCGA PRAD (prostate adenocarcinoma) dataset was downloaded, differentially expressed genes (DEGs) were analyzed. Subsequently, the enrichment analysis was performed on the DEGs. All the mentioned steps were performed with the TCGAbiolinks R package [27,28]. After that, the rpart R module (version 4.1.19) [29,30] was used to perform recursive partitioning and the progression-free survival analysis. Furthermore, the R commander (version 2.8-0) and EZR packages (version 1.61) [31,32] were used to establish the Kaplan–Meier estimate of individual nodes determined by rpart. The reason why we performed survival analysis with all the matrisome and adhesome genes and not only DEGs is that rpart analysis defines risk subgroups, so the changes of gene expressions in a subgroup of patients could be masked by global levels of gene expression in pooled prostate cancer samples.

### 2.1. ECM- and IACs-Related Genes’ Retrieval

Matrisome is the ensemble of genes encoding the extracellular matrix (ECM) and ECM-associated proteins, which was predicted bioinformatically in the genome of various model organisms by using the characteristic domain-based organization of ECM proteins [33,34]. The matrisome genes (N = 1027) were retrieved from: http://matrisome.org/ (accessed on 1 September 2022) [33,34]. These genes can be further divided into genes encoding core matrisome proteins and matrisome-associated proteins.

The adhesome network is a literature-based network that is composed of known cellular components, constituting the focal adhesion complex in mammalian cells [35,36]. The adhesome genes (N = 232) were retrieved from: https://adhesome.org/ (accessed on 1 September 2022) [35,36].

The consensus adhesome consists of the 60 most common proteins that are extracted from quantitative proteomic datasets, in which IACs were induced by the canonical ligand fibronectin. These proteins are likely to represent the core cell adhesion machinery and were retrieved from [37].

The final combined list of matrisome, adhesome, and consensus adhesome genes had 1286 genes in total, and is provided in the Supplementary Material.

### 2.2. Data Preparation

The TCGAbiolinks R package [27,28] was used to download, prepare, and analyze The Cancer Genome Atlas (TCGA) [38] prostate adenocarcinoma (PRAD) dataset. This dataset contains gene expression data for 497 prostate cancer patients and corresponding non-transformed prostate tissues for a subset of 52 patients. The same R package was used to pre-process, normalize, and filter the dataset and prepare it for the differential gene expression, functional enrichment, and survival analyses.

### 2.3. Differential Gene Expression and Functional Enrichment Analyses

To gain insight into differentially expressed genes (DEGs) in prostate cancer in comparison to non-transformed prostate tissue, we set the following criteria in the TCGAbiolinks R package: |log2FC| ≥ 1 (corresponding to |fold change| ≥ 2) and FDR (false discovery rate) *p*-value < 0.01. These conditions yielded 2037 DEGs. Among these 2037 genes, we singled out ECM- and IACs-related genes with changed expression in prostate cancer.

The functional enrichment analysis for the Gene Ontology Cellular Component (GO CC) category using 2037 DEGs was performed by using the TCGAbiolinks R package.

### 2.4. Clinical Data Retrieval

The clinical data in Table 1 were downloaded from the cBioPortal [39] and NCI Genomic Data Commons (GDC, TCGA) portals [40]. The downloaded data were combined in a single file according to the patients’ unique TCGA codes. In total, there were 493 patients with clinical information available. The event that we considered was progression-free survival (PFS, N = 93). This is because, fortunately, only a smaller percentage of patients had an event needed for overall survival analyses. This makes an overall survival analysis in prostate cancer suboptimal. Some variables in our analysis contained missing data. However, the decision trees that we obtained in the survival analysis by using recursive partitioning hold an advantage in comparison to traditional statistical methods as they are not as affected by missing data [41].

### 2.5. The Survival Analysis

Variables from Table 1 (age, Gleason score, TNM staging, and residual tumor information) were supplemented with gene expression data for matrisome and adhesome genes, and their prognostic value was determined through recursive partitioning. The American Joint Committee on Cancer (AJCC) recommends recursive partitioning for the analysis in prognostic studies [42,43]. We used the rpart package [29,30] in the programming language R (version 4.2.1) [44] for the creation of survival trees. Rpart is an abbreviation for Recursive PARTitioning, and it is the frequently used method for the construction of survival trees. Survival trees obtained through the rpart method enable visual inspection and comparison of prognostic factors [42,43]. The basic principles of the rpart method are elaborated more closely in our previous publications [26,45]. Briefly, first we calculated the importance of individual variables. Second, we generated the survival tree, which is defined by its decision nodes and terminal nodes (leaves). The analysis began with all patients, who were then further divided into prognostic subgroups at each decision node. At the first decision node (the root node), a logical check was conducted. If the criterion imposed by that node was met, the left side of the tree was followed, and if not, the right side was followed. This action was repeated at each decision node through to the point at which the terminal node was reached. At each decision node, a variable was used to subdivide patients in two subgroups, with maximum differences in their hazard ratios (HR). If no further improvement in subdivision was possible, the terminal nodes were reached. Patients in the first decision node (the root node) had a hazard ratio of 1, and the hazard ratio for patients in each further node was assigned in comparison to this value. Overfitting is a frequent problem in machine learning which, in this case, can lead to an extensive fragmentation of the tree, for which it is hard to find a biological meaning. To avoid overfitting, we set the complexity parameter (CP) to 0.0592 and 0.0636 for the ECM and for the IAC genes, respectively.

The log-rank test was used to analyze the difference in survival between patients in terminal nodes, and the results were presented as survival curves showing the Kaplan–Meier survival estimate [46]. The analysis was performed by using the EZR package [32], an add-on in R commander (a basic-statistics graphical user interface to R) [31]. The obtained data were statistically significant since the log-rank test *p*-value was <0.001.

## 3. Results

### 3.1. The Expression of Matrisome and Adhesome Genes Appears to Be Aberrant in Prostate Cancer

**Figure 2:**
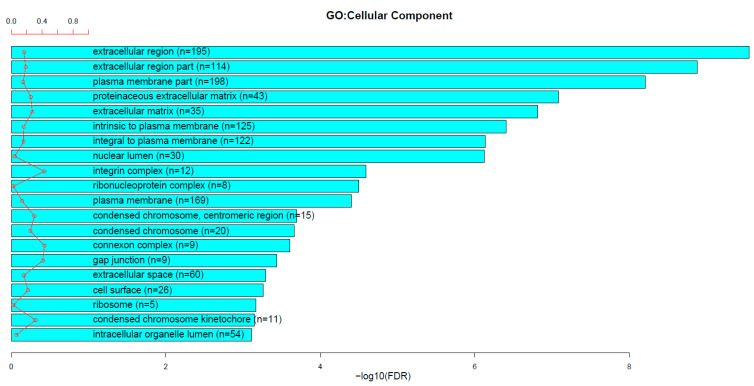
The enrichment analysis of differentially expressed genes (N = 2037) in prostate cancer. The TCGA PRAD dataset was used with the following criteria: |log2FC| ≥ 1 (corresponding to |fold change| ≥ 2) and FDR (false discovery rate) *p*-value < 0.01. The enrichment analysis was performed by using the TCGAbiolinks R programming language package.

Gene expression analysis of prostate tissue from prostate cancer patients described in the Materials and Methods Section revealed 2037 differentially expressed genes (DEGs) when compared to non-transformed prostate tissue. The result of the functional enrichment analysis for the Gene Ontology Cellular Component (GO CC) category using these 2037 genes and the TCGAbiolinks R package is provided in Figure 2. The top-20 GO Cellular Compartment terms are shown. The enrichment analysis on these genes showed that the GO terms ‘extracellular matrix’ (N = 35 genes) and ‘integrin complex’ (N = 12 genes) were among those that were highly enriched in the Gene Ontology Cellular Component (GO CC) category (Figure 2). In the GO Biological Process (GO BP) category, we detected the ‘cell adhesion’ term (N = 62 genes) among the top-20 categories. The ECM- and IACs-related DEGs are listed in Table 2.

Among the ECM- and IACs-related DEGs are many proteins that give a structure to the ECM, such as collagens, various ECM glycoproteins, and ECM proteoglycans (Table 2). Additionally, the expression of ECM regulators, involved in organization of the ECM, is also perturbed. The genes encoding for secreted factors that stimulate the crosstalk between tumor and host cells, (lymph)angiogenesis, and the hijack and recruitment of immune cells, also change expression. With such an extensive perturbation in the ECM composition, it is hard to speculate which characteristics of the ECM changed. However, it is known from the literature that the tumor ECM in general gains an increase in density and mechanical stiffness [47] due to the changed quantity of ECM structural proteins and the extent of crosslinking.

Integrins are a link between the ECM and intracellular machinery that are highly alerted to the changes in the ECM. It is interesting to note that in prostate cancer, integrins and adhesome genes mainly show decreased expression (Table 2). It would be important to relate these differences to phenotypes of prostate cancer and to decipher whether there are compensatory mechanisms, such as, for example, the increase in the expression of some of the integrin ligands (e.g., collagens).

The expression of genes that we showed are involved in the prognosis of PFS of prostate cancer patients, *PTPN2* and *FMOD*, did not change the global expression between prostate cancer and non-transformed tissue according to the criteria used (|log2FC| ≥ 1 and FDR *p*-value < 0.01).

### 3.2. ECM- and IACs-Related Genes Are Involved in Prognosis of Progression-Free Survival in Prostate Cancer Patients

**Figure 3:**
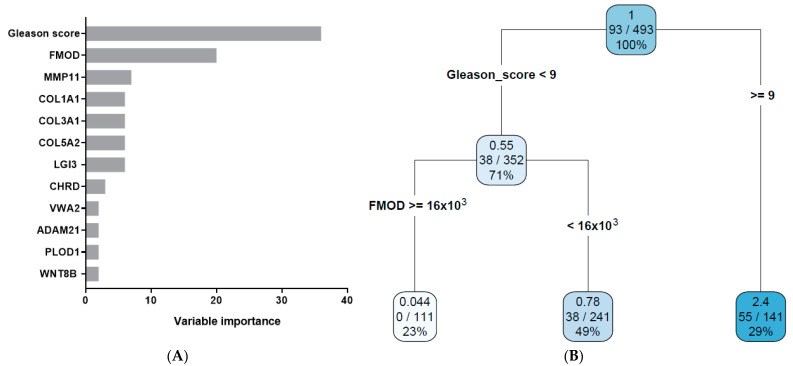
Variable importance (**A**) and the decision tree (**B**) for the ECM-related genes. Darker color denotes a higher risk for progression. The initial number in the decision node rectangle stands for the hazard ratio (HR), and the numbers in the second row denote patients with an event (progression) vs. the total number of patients. The number in the third row equals the percentage of patients belonging to that node.

**Figure 4:**
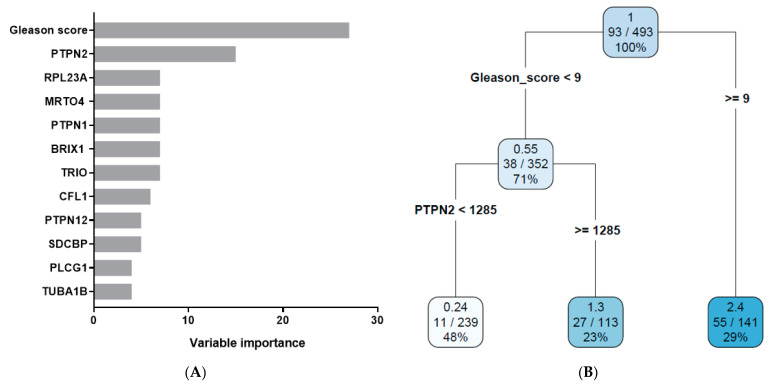
Variable importance (**A**) and the decision tree (**B**) for the IACs-related genes. Darker color denotes a higher risk for progression. The initial number in the decision node rectangle stands for the hazard ratio (HR), and the numbers in the second row denote patients with an event (progression) vs. the total number of patients. The number in the third row equals the percentage of patients belonging to that node.

Recursive partitioning is the method recommended by the AJCC for the analysis of prognostic studies [42,43]. Therefore, we used the rpart method to determine the prognostic value of the following variables (Table 1): age, Gleason score, TNM staging, residual tumor information, and the gene expression data for the ECM- and IACs-related genes. The ECM- and IACs-related genes were separately analyzed. The importance of individual variables is shown in Figure 3A and Figure 4A. By performing the rpart analysis, our result from a previous publication, which found the Gleason score to be the strongest prognostic factor in prostate cancer among the studied variables, was confirmed [26]. The five most informative variables in Figure 3A in addition to the Gleason score were the expressions of *FMOD*, *MMP11*, *COL1A1*, *COL3A1*, and *COL5A2* genes. Among them, only *FMOD* emerged on the survival tree. In Figure 4A, the five most informative variables in addition to the Gleason score were the expressions of *PTPN2*, *RPL23A*, *MRTO4*, *PTPN1*, and *BRIX1*. Among those, only the *PTPN2* gene expression variable emerged on the survival tree. From the variable importance analysis, it was evident that even the most informative individual variable (the Gleason score) had a score of only 36 (matrisome data) and 27 (adhesome data) in comparison to the whole model, bearing the score of 100. Therefore, the multivariate approach to survival analysis is the only way to correctly describe the patients’ prognosis.

AJCC guidelines for prognostic studies suggest that a prognostic value of a single variable is evaluated by considering the other variables [42,43]. The rpart method follows this criterion because rpart uses all variables in the analysis. The results of the rpart algorithm performed on our data are presented on a survival tree (Figure 3B and Figure 4B). Figure 3B and Figure 4B show that, by using two variables in each survival tree, patients were further subdivided into two decision nodes and three terminal nodes (leaves) in each tree.

Variables used in the decision nodes in Figure 3B and Figure 4B are the Gleason score and the *FMOD* and *PTPN2* gene expressions. *FMOD* and *PTPN2* refined the prognosis of patients with a Gleason score < 9, respectively. The importance of the variables in Figure 3B and Figure 4B was determined by their position in the survival tree: the topmost variable (the Gleason score) holds the largest amount of information, the variable below the topmost is the second largest by the content of information, and so on. It is obvious from Figure 3B and Figure 4B that there were three prognostic subgroups on each. For Figure 3B, they were: (a) low Gleason score and high *FMOD* expression, (b) low Gleason score and low *FMOD* expression, and (c) high Gleason score. The HR gradually increased from the left to the right of the survival tree. By using the complexity parameter (CP) = 0.0592, we did not find a variable that further refined the high Gleason score patients (≥9). However, when the CP was set at CP = 0.0371, we obtained a separation in that group of patients according to the expression of the *MFAP3* gene. Namely, *MFAP3* high expression (≥1389) was associated with worse survival (HR 2.8 vs. 0.37). In Figure 4B, we also established three prognostic subgroups: (a) low Gleason score and low *PTPN2* expression, (b) low Gleason score and high *PTPN2* expression, and (c) high Gleason score. In this survival tree, the HR also gradually increased from the left to the right.

To conclude, by using the Gleason score information supplemented with the expression of *FMOD* and *PTPN2* genes, a stratification of prostate cancer patients into several prognostic subgroups with significantly different hazard ratios (low, medium, and high risk of progression) was achieved.

**Figure 5:**
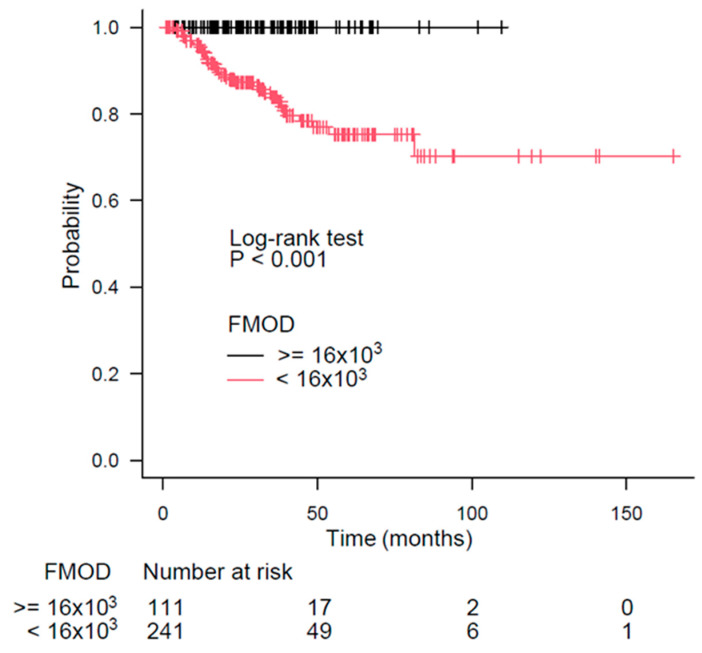
Difference in patients’ survival for the left and the right branches of the second decision node from Figure 3B, which uses the *FMOD* gene expression as a separation criterion.

**Figure 6:**
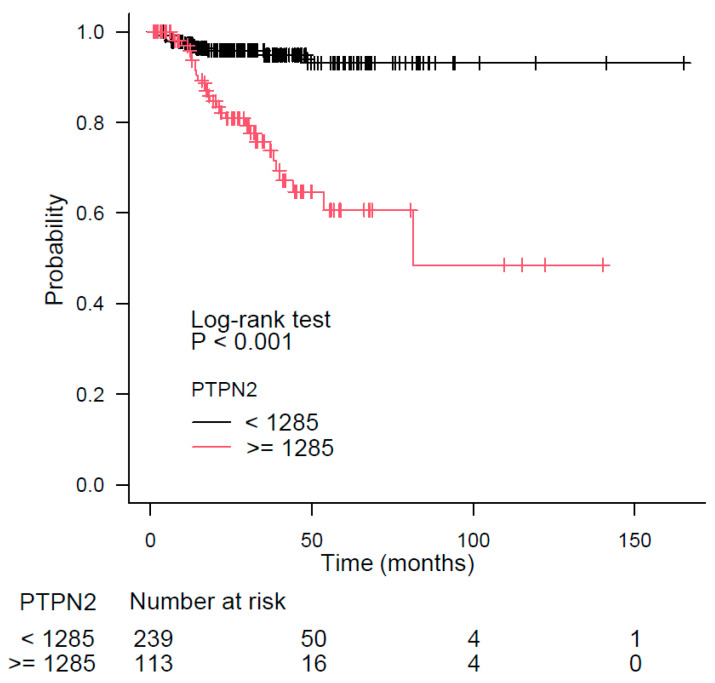
Difference in patients’ survival for the left and the right branches of the second decision node from Figure 4B, which uses the *PTPN2* gene expression as a separation criterion.

The results of recursive partitioning (Figure 3B and Figure 4B) were further supplemented by survival curves obtained using the Kaplan–Meier method for subgroups from each decision node. The difference in survival for subgroups defined by the left and the right branches of the decision node 1 (the Gleason score) is shown in our previous publication [26]. The subgroups from decision node 2 are shown in Figure 5 (*FMOD* expression) and Figure 6 (*PTPN2* expression). The log-rank test *p*-value was statistically significant (*p* < 0.001) for both genes (Figure 5 and Figure 6).

## 4. Discussion

The driving processes in prostate cancer progression encompass intertwined actions of several signaling pathways, which are potentiated by genetic and epigenetic alterations, changes in gene expression, and post-transcriptional and post-translational modifications [1,2,48]. However, although a large amount of data exists regarding the mentioned processes, one of the greatest barriers in prostate cancer treatment is still the inability to precisely foresee the course of a disease, and therefore, to define the risk subgroups which would guide the treatment options. In our previous work, we added to the efforts which try to reveal prostate cancer PFS prognosis biomarkers [26]. In that work, the Gleason score emerged as the most informative prognostic factor among all the clinical and the gene expression variables studied. Herein, we extended the analysis to the ECM- and IACs-related genes. Our results are based on the TCGA PRAD dataset, and they dissect differential expression of ECM- and IACs-related genes and their value as prognostic factors in the progression-free survival of prostate cancer patients.

### 4.1. Extracellular Matrix-Related Genes’ Expression and Prognostic Significance in Progression-Free Survival of Prostate Cancer Patients

The ECM is emerging among the main determinants of tumor growth and dissemination [49,50]. Therefore, it does not come as a surprise that its components bear prognostic and therapeutic value in many different cancer types [11]. The numerous examples include: breast cancer [51], metastatic melanoma [52], gastric cancer [53], acute myeloid leukemia [54], non-small-cell lung cancer [55], glioblastoma [56], colon cancer [57], hepatocellular carcinoma [58], esophageal squamous cell carcinoma [59], and renal clear cell carcinoma [60].

In this article, based on the TCGA PRAD dataset, ECM (matrisome) gene expression appeared to be highly aberrant in prostate cancer tissue. The enrichment analysis on the DEGs showed that the GO term ‘extracellular matrix’ (N = 35 genes) was among those that were enriched in the Gene Ontology Cellular Component (GO CC) category (Figure 2). Genes from all the ECM categories (Table 2) showed changed expression. As mentioned in the Results Section, with such a comprehensive change in the expression of individual components, it is hard to speculate which of the ECM general characteristics are changed in prostate cancer. However, it is known from the literature that the cancers’ ECMs in general gain an increase in density and mechanical stiffness [47].

In a search for prognostic factors among the ECM-related genes, the expression of the *FMOD* gene appeared to refine the prognosis based on the Gleason score. Namely, the patients with a Gleason score lower than 9 were further subdivided into two prognostic subgroups based on the *FMOD* gene expression. The patients with high *FMOD* expression had better survival (Figure 5). The *FMOD* gene encodes the fibromodulin protein, which belongs to the family of small interstitial proteoglycans [61]. This protein interacts with type I and type II collagen fibrils and inhibits fibrillogenesis in vitro. Therefore, fibromodulin may play a role in the assembly of the extracellular matrix. It may also regulate TGF-beta activities by sequestering TGF-beta into the extracellular matrix (www.genecards.org accessed on 1 December 2022). In the prostate cancer setting, FMOD was shown to be overexpressed in human prostate epithelial cancer cell lines in vitro. Additionally, the authors showed that the cancerous tissue expressed significantly higher levels of intracellular fibromodulin compared to matched, benign tissue from the same patients. Higher levels were also detected in cancerous tissue in comparison to tissue from patients with only a benign disease [62,63]. Furthermore, in a study based on Brazilian individuals, *FMOD* gene variants were suggested to be potential biomarkers for prostate cancer and benign prostatic hyperplasia [64]. However, in a recent article, it was shown that higher *FMOD* expression was associated with better disease-free survival of prostate cancer patients, a finding that agrees with our results [65]. This would mean that, although the cancerous tissue has higher *FMOD* expression than non-transformed prostate tissue, in prostate cancer, higher *FMOD* expression bears a better prognosis. Here, it needs to be remembered that, besides *FMOD*, our analysis showed that *COL1A1*, *COL3A1*, and *COL5A2* genes were also shown to have high informative value in the prognosis of PFS when individually analyzed (Figure 3A). It would be interesting to imply their functional role and to further delineate whether FMOD and these three collagen genes are interacting in the architecture of certain prostate cancer phenotypes that affect the patients’ survival.

### 4.2. Integrin Adhesion Complexes-Related Genes Expression and Prognostic Significance in Progression-Free Survival of Prostate Cancer Patients

Integrin receptors are involved in almost every process of cancer formation and progression [66]. Therefore, it is not surprising that numerous preclinical studies on targeting integrins in different cancer types revealed encouraging results. However, there are still obstacles in translating these results into the clinics [67,68]. In addition to all the difficulties [69,70], in our recent paper [19], we suggested that integrin crosstalk could potentially complicate and undermine the effects of targeting integrins. Integrin crosstalk is a phenomenon in which the modulation of the activity and/or expression of one integrin (subunit or a heterodimer) affects the activity and/or expression of other integrin(s) (subunit(s) or heterodimer(s)). To circumvent integrin crosstalk, but to keep the advantages of targeting the integrin pathway, we suggest that the analysis of proteins downstream of integrin ligation and activation could reveal effective therapeutic targets. Therefore, in this paper, we focused on integrin adhesion complexes (IACs), in a search for potential prognostic biomarkers and therapeutic targets in prostate cancer. IACs are essential protein-composed adhesion structures whose components were also detected outside of Metazoa, confirming their ancient evolutionary origin [71]. There are several types of IACs recognized [21], which include nascent adhesions [72], focal complexes [73], focal adhesions [74], fibrillar adhesions [75], reticular adhesions [76], and hemidesmosomes [77]. Although IACs vary in their appearance, size, dynamics, and composition, the core components of integrin adhesome have been identified by several groups [35,36,37]. The integrin adhesome consists of proteins that are affiliated with the structure and signaling activity of integrin-mediated adhesions [36]. By analyzing the core integrin adhesome components, we found that their expression is highly perturbed in prostate cancer. Namely, the category ‘integrin complex’ appeared among the top functionally enriched Gene Ontology Cellular Component (GO CC) terms (Figure 2). Furthermore, we detected 44/264 (16.7%) adhesome genes whose expression was significantly changed by ≥2 times (either up- or down-regulated) in prostate cancer, in comparison to non-transformed prostate tissue (Table 2). An important notion is that majority of these genes are downregulated in the prostate cancer tissue. Their functional role and the potential compensatory mechanisms remain to be investigated.

In addition to changes in gene expression, in our analysis, we found that the expression of some of the adhesome genes was correlated with PFS in univariate and multivariate approaches. The examples of genes implicated in the univariate approach are *PTPN2*, *RPL23A*, *MRTO4*, *PTPN1*, and *BRIX1* (Figure 4A). However, except for *PTPN2*, those genes did not emerge on the survival tree. This would mean that the expression of the mentioned genes is probably correlated with some of the variables which already hold a prognostic value, such as, for example, the Gleason score. It is interesting to note that three genes (*PTPN1*, *PTPN2*, and *PTPN12*) from the PTPN family of protein tyrosine phosphatases emerged in univariate analysis. Tyrosine phosphorylation is an important post-translational modification in cell adhesion that is dynamically regulated by the protein tyrosine phosphatases and kinases [78]. While PTPN1 [79,80,81] and PTPN12 [82,83] were implicated in prostate cancer biology, the involvement of PTPN2 in prostate cancer is not documented [84]. Regarding integrin signaling, complex roles for PTPN1 [85,86,87,88], PTPN2 [89], and PTPN12 [90] have been documented. Despite this, it needs to be mentioned that PTPN proteins have other, broader roles [84]. Therefore, it cannot be ruled out that some of these other roles are also important for the biology of prostate cancer.

The *PTPN2* gene expression appeared on the survival tree as a variable that refines the PFS of lower (<9) Gleason score patients. Our results suggested that its higher expression bears a poorer prognosis. PTPN1 and PTPN2 are highly related PTPs [84], but, as mentioned previously, PTPN2 has not been implicated in prostate cancer. However, *PTPN2* is a key predictor of prognosis for pancreatic adenocarcinoma, and its higher expression is associated with a poor prognosis [91]. Overexpression of *PTPN2* also predicted a poor survival in clear cell renal cell carcinoma [92], which agrees with our results. However, low *PTPN2* expression was associated with poor overall survival in ovarian serous cystadenocarcinoma [93], indicating its versatile roles in different cancer types. The connection of PTPN2 with integrin signaling was confirmed by several articles, which indicate activation of PTPN2 by integrins. Namely, it was recently shown that the catalytic activity of PTPN2 is auto-regulated by its intrinsically disordered tail and activated by ITGA1 [89]. An earlier article also documented that PTPN2 is activated by the integrin ITGA1/ITGB1 and that it subsequently dephosphorylates EGFR and negatively regulates EGF signaling [94]. In line with this, the same group showed that PTPN2 activity was induced upon integrin-mediated binding of endothelial cells to the collagen matrix [95]. However, the potential role of PTPN2 activation by integrins in prostate cancer remains to be investigated. To conclude, PTPN2 might be a potential target in prostate cancer treatment, whose targeting is achievable because the PTPN2 inhibitors are available.

An interesting notion is that neither ECM- nor IACs-related genes defined risk subgroups for the Gleason score ≥ 9, according to the conservative complexity parameters that we selected. It could be that the high Gleason score cancers show such aberrant ECM- and IACs-related genes’ expression that are of a great importance for cancer progression and, therefore, are common to all patients. This would mean that ECM- and IACs-related genes’ aberrant expression is underlying for all high Gleason score (≥9) patients.

### 4.3. Methodological Considerations

In this article we used recursive partitioning to define the risk subgroups of prostate cancer patients in the analysis that included clinical information and the gene expression data. Recursive partitioning is the method recommended by AJCC for the analysis of prognostic studies [42,43]. Due to the prostate cancer heterogeneity, it is to be expected that this method better describes its diversity than the Cox regression analysis, which is used by majority of papers dealing with similar questions. Moreover, the survival tree, obtained by recursive partitioning, is easier to interpret than the Cox regression results. Therefore, we believe that our approach is more appropriate to analyze the prostate cancer survival data.

## 5. Conclusions

ECM is the first frontier of the cell towards its surroundings, and it is among the main determinants of the cell’s behavior. Therefore, important roles of the ECM in cancer development, progression, and prognosis were documented. By using the TCGA PRAD dataset, in this article, the expression of ECM genes in prostate cancer was analyzed and correlated with progression-free survival of prostate cancer patients. We revealed that the expression of ECM-related genes changed in prostate cancer. Moreover, the ECM-related genes showed prognostic significance for the prostate cancer patients, who were stratified according to the Gleason score. Our results confirmed the important roles for the ECM-related genes in prostate cancer and suggested the potential biomarkers of prostate cancer progression from the list of the ECM-related genes.

Integrins are among the main receptors for the ECM ligands. Several unique characteristics, including integrin crosstalk and the formation of IACs, make integrins exceptional among the signaling receptors. Therefore, their roles in tumor formation, progression, and drug resistance were noted early on [96]. In this paper, we showed that the expression of integrin and IAC genes changed in prostate cancer. Moreover, some of these genes are appearing in univariate and multivariate approaches in the prognosis of PFS, suggesting their potential role in the discovery of biomarkers of prostate cancer progression. Consequently, our results support the early notion that considered integrins (and downstream proteins) attractive therapeutic targets, a strategy that is still hotly debated [68,70].

## Figures and Tables

**Table 1:** Clinical information of The Cancer Genome Atlas patients. The number (N) and the percentage (in parentheses) of patients that belong to a certain category are shown. Some categories contain unknowns (NAs). The table was modified and adapted from our recent publication [26].

		No Progression	Progression
***n*** **, total**		400 (100%)	93 (100%)
Age, years	<60	166 (41.5%)	34 (36.6%)
	≥60	234 (58.5%)	59 (63.4%)
Gleason score	6	44 (11%)	1 (1.1%)
	7	221 (55.3%)	24 (25.8%)
	8	49 (12.3)	13 (14%)
	9	84 (21%)	53 (57%)
	10	2 (0.5%)	2 (2.2%)
Clinical T stage	cT1	158 (39.5%)	17 (18.3%)
	cT2	137 (34.3%)	35 (37.6%)
	cT3	28 (7%)	24 (25.8%)
	cT4	1 (0.3%)	1 (1.1%)
	NA	76 (19%)	16 (17.2%)
Clinical M stage	cM0	362 (90.5%)	89 (95.7%)
	cM1	2 (0.5%)	1 (1.1%)
	NA	36 (9%)	3 (3.2%)
Pathologic T stage	pT2	172 (43%)	14 (15.1%)
	pT3	215 (53.8%)	75 (80.7%)
	pT4	7 (1.8%)	3 (3.2%)
	NA	6 (1.5%)	1 (1.1%)
Pathologic N stage	pN0	280 (70%)	62 (66.7%)
	pN1	56 (14%)	22 (23.7%)
	NA	64 (16%)	9 (9.7%)
Residual tumor	R0	266 (66.5%)	46 (49.5%)
	R1	102 (25.5%)	44 (47.3%)
	R2	5 (1.3%)	0
	RX	13 (3.3%)	2 (2.2%)
	NA	14 (3.5%)	1 (1.1%)

**Table 2:** Matrisome and adhesome genes up- (red; N = 71) and down-regulated (green; N = 177) in prostate cancer in comparison to healthy prostate tissue according to TCGA PRAD data. The numbers in parentheses represent the fold change (FC; threshold |FC| ≥ 2x). The adjusted *p*-value is <0.01 for each gene. The genes are shown in descending FC values’ order. ECM glycoproteins, collagens, and proteoglycans belong to the core matrisome category, and ECM-affiliated proteins, ECM regulators, and secreted factors belong to the category of matrisome-associated proteins [33,34].

Category	Genes (FC)
Integrins	***ITGAX*** (2.1), ***ITGA7*** (0.5), ***ITGA1*** (0.5), ***ITGA5*** (0.4), ***ITGB8*** (0.4), ***ITGB4*** (0.4), ***ITGA8*** (0.4), ***ITGA2*** (0.4), ***ITGA9*** (0.4), ***ITGB6*** (0.4), ***ITGB3*** (0.4).
Adhesome	***PDLIM5*** (3.5), ***TSPAN1*** (2.2), ***SRCIN1*** (2.0), ***INPP5D*** (0.5), ***TES*** (0.5), ***PLS3*** (0.5), ***DNAJB1*** (0.5), ***JUB*** (0.5), ***CALD1*** (0.5), ***HSPB1*** (0.4), ***NRP2*** (0.4), ***VAV3*** (0.4), ***KCNH2*** (0.4), ***PEAK1*** (0.4), ***LPP ***(0.4), ***PRKCA*** (0.4), ***NEXN*** (0.4), ***FERMT2*** (0.4),***TRIP6***(0.4), ***CSRP1*** (0.4), ***FLNA*** (0.4), ***PDLIM7*** (0.4), ***VCL*** (0.4), ***SVIL*** (0.4), ***PALLD ***(0.4), ***LIMS2*** (0.4), ***TNS1*** (0.4), ***TGFB1I1*** (0.3), ***SORBS1*** (0.3), ***CAV1*** (0.3), ***LDB3*** (0.3), ***SYNM*** (0.3), ***FLNC*** (0.3).
ECM Glycoproteins	***FGB*** (43.1), ***FGL1*** (12.4), ***SLIT1*** (9.2), ***ZP1*** (6.4), ***VWA5B1*** (6.0), ***CTHRC1*** (4.3), ***COMP*** (4.0), ***SPON2*** (3.5), ***THBS4*** (3.2), ***ZP3*** (2.5), ***LGI1*** (2.4), ***NTN5*** (2.0), ***EMILIN1*** (0.5), ***NTN4*** (0.5), ***FBLN1*** (0.5), ***THSD4*** (0.5), ***MATN2*** (0.5), ***NID1*** (0.5), ***NTN1*** (0.5), ***FN1*** (0.5), ***SPARCL1*** (0.5), ***RSPO2*** (0.5), ***VWA5A*** (0.4), ***EMILIN3*** (0.4), ***GAS6*** (0.4), ***BMPER ***(0.4), ***RSPO3*** (0.4), ***SLIT3*** (0.4), ***EFEMP2*** (0.4), ***VTN*** (0.4), ***NDNF*** (0.4), ***MFAP5*** (0.4), ***TINAGL1*** (0.4), ***EDIL3*** (0.4), ***EFEMP1*** (0.4), ***LGI3*** (0.3), ***VIT*** (0.3), ***SPON1*** (0.3), ***NELL2*** (0.3), ***SMOC1*** (0.3), ***VWCE*** (0.3), ***LAMB3*** (0.3), ***DPT*** (0.3), ***GLDN*** (0.3), ***WISP2*** (0.3), ***SBSPON*** (0.2), ***DMBT1 ***(0.2), ***VWA5B2*** (0.2).
Collagens	***COL2A1*** (15.1), ***COL10A1*** (5.9), ***COL11A1*** (5.6), ***COL9A2*** (3.5), ***COL28A1*** (2.6), ***COL11A2*** (2.4), ***COL12A1*** (2.0), ***COL21A1*** (0.4), ***COL9A1*** (0.4), ***COL23A1*** (0.3), ***COL4A6*** (0.3), ***COL17A1*** (0.3).
Proteoglycans	***ASPN*** (4.5), ***ACAN*** (2.8), ***HAPLN2*** (0.5), ***HAPLN1*** (0.4), ***OGN*** (0.4), ***SPOCK3 ***(0.4).
ECM-Affiliated Proteins	***SFTPA2*** (4.3), ***GPC2*** (4.3), ***CLEC18A*** (4.2), ***HPX*** (3.5), ***REG4*** (3.5), ***MUC13*** (3.1), ***C1QTNF3*** (3.0), ***MUC2*** (2.4), ***CSPG5*** (2.4), ***COLEC12*** (2.3), ***ELFN2*** (2.2), ***SEMA3D*** (0.5), ***SEMA7A*** (0.5), ***ANXA2*** (0.5), ***SEMA3B*** (0.5), ***SDC1*** (0.5), ***SEMA5A*** (0.5), ***CLEC3B*** (0.5), ***PARM1*** (0.4), ***ANXA6*** (0.4), ***ANXA8L1*** (0.4), ***CSPG4*** (0.4), ***PLXNA4*** (0.4),***CLEC3A***(0.3), ***SEMA3A*** (0.3), ***ANXA8*** (0.3), ***C1QTNF1*** (0.3), ***SEMA6D*** (0.3), ***LGALS4*** (0.3), ***MUC4*** (0.2), ***C1QL1*** (0.2), ***MUC15*** (0.2), ***ANXA9*** (0.1), ***MUCL1*** (0.1), ***MUC6 ***(0.0), ***ANXA13 ***(0.0).
ECM Regulators	***CST2*** (13.3), ***MMP26*** (12.8), ***CST1*** (9.8), ***ADAM2*** (8.3), ***SERPINA11*** (5.9), ***HABP2*** (5.4), ***TGM3*** (5.1), ***ADAM21*** (5.0), ***F12*** (3.9), ***PCSK6*** (3.7), ***MMP9*** (2.7), ***MMP10*** (2.6), ***MMP11*** (2.5), ***ITIH4*** (2.5), ***ADAM32*** (2.2), ***ADAMDEC1*** (2.1), ***MMP12*** (2.0), ***TIMP4 ***(0.5), ***LEPREL2*** (0.5), ***F10*** (0.5), ***SERPINB1*** (0.5), ***EGLN3*** (0.5), ***SERPINB3*** (0.5), ***ADAMTSL4*** (0.5), ***SERPINF1*** (0.5), ***SERPINB11*** (0.5), ***PRSS12*** (0.4), ***ITIH5*** (0.4), ***TIMP3*** (0.4), ***PAPPA*** (0.4), ***CPAMD8*** (0.4), ***MASP1*** (0.4), ***ADAM7*** (0.4), ***TGM1*** (0.4), ***SERPINA4*** (0.3), ***SERPINA1*** (0.3), ***SERPINF2*** (0.3), ***ADAMTS5*** (0.3), ***SERPINB5*** (0.3), ***TGM4*** (0.2), ***CST6*** (0.2), ***KY*** (0.2), ***CST4*** (0.1), ***SLPI*** (0.1), ***SERPINA5*** (0.0).
Secreted Factors	***ANGPTL3*** (25.1), ***AMH*** (13.9), ***GDF1*** (6.7), ***CCL18*** (5.3), ***C1QTNF9B*** (4.5), ***GDF15*** (3.1), ***SFRP4*** (3.1), ***CXCL11*** (3.1), ***CXCL14*** (2.5), ***CXCL10*** (2.4), ***CCL22*** (2.3), ***EGF*** (2.3), ***CXCL9*** (2.3), ***INHA*** (2.3), ***WFIKKN1*** (2.1), ***FGF14*** (2.1), ***CHRD*** (2.1), ***FGFBP3*** (2.0), ***FGF2 ***(0.5), ***S100A9*** (0.5), ***CHRDL2*** (0.5), ***S100A2*** (0.5), ***ANGPTL4*** (0.5), ***PDGFD*** (0.5), ***LEFTY1*** (0.5), ***CSF3*** (0.4), ***FGF7*** (0.4), ***KITLG*** (0.4), ***BDNF*** (0.4), ***TGFB3*** (0.4), ***S100A16*** (0.4), ***AREG*** (0.4), ***S100B*** (0.4), ***NTF4*** (0.4), ***WNT3A*** (0.4), ***S100A6*** (0.4), ***NRG1*** (0.4), ***IL1RN*** (0.4), ***SCUBE3*** (0.4), ***CXCL13*** (0.4), ***CRHBP*** (0.4), ***WNT2B*** (0.4), ***CTF1*** (0.4), ***WNT10A*** (0.3), ***FGFBP1*** (0.3), ***SCUBE1*** (0.3), ***NRG2*** (0.3), ***PGF*** (0.3), ***ANGPTL1*** (0.3), ***CHRDL1*** (0.3), ***ANGPT1*** (0.3), ***CCBE1*** (0.3), ***GDF10*** (0.2), ***S100A14*** (0.2), ***WIF1*** (0.2), ***BMP5*** (0.2), ***SFRP5*** (0.1).

## Data Availability

In this article, we used The Cancer Genome Atlas (TCGA) prostate adenocarcinoma (PRAD) dataset, available at: https://gdc.cancer.gov/ (accessed on 1 September 2022).

## Supplementary Materials

The following supporting information can be downloaded at: https://www.mdpi.com/article/10.3390/biomedicines11072006/s1, Table S1: A list of ECM- and IACs-related genes used in this study.
